# A Web-Based Supportive Intervention for Families Living With Depression: Content Analysis and Formative Evaluation

**DOI:** 10.2196/resprot.3051

**Published:** 2014-02-14

**Authors:** Sigrid Stjernswärd, Lars Hansson

**Affiliations:** ^1^Department of Health SciencesLund UniversityLundSweden

**Keywords:** family caregivers, depression, online social networks, social support

## Abstract

**Background:**

Relatives of people with a mental illness who live together can experience additional burdens that may require support. A Web-based tool including a psychoeducation module, a diary, and a password-protected forum was developed to support relatives of a person with depression.

**Objective:**

The objective of our study was to explore participants’ use of the Web-based tool, with focus on the forum, and to assess its potential health and psychosocial benefits.

**Methods:**

Twenty-five people participated in this explorative open trial. Self-rating instruments assessing caregiver burden, stigma, and the tool’s usability were analyzed with Carer QoL7-D, DISC-12, and a system usability scale. A summary measure of subjective burden was assessed with CarerQoL-VAS. The forum posts were studied using content analysis.

**Results:**

The majority reported fulfillment from their caregiving tasks (84%, 21/25), and had relational problems (76%, 19/25), their own mental health problems (72%, 18/25), support (72%, 18/25), and difficulties coordinating daily activities with caregiving (56%, 14/25). Most (72%, 18/25) reported having been able to use their inner strength to cope with stigma and discrimination, 64% (16/25) had concealed or hidden the person’s condition, and 40% (10/25) reported having been avoided or shunned by people who knew about the illness. Forty-eight percent (12/25) reported unfair treatment from family; 40% (10/25) in marriage or divorce and 36% (9/25) from mental health staff. Almost one-third (28%, 7/25) reported having stopped themselves from having a close personal relationship. Participants’ subjective assessment of the tool’s usability resulted in a mean of 61.5 (range, 22.5-90; possible total value 0-100; >70=good). Ten people participated in the forum; content analysis resulted in five categories describing relatives’ situations: balancing the caregiver’s role and relationship to the patient; their own lives and need for support; resources and patient advocacy; a looming shadow on leisure, social, and professional life; and interaction and social support.

**Conclusions:**

Further studies are needed to explore optimal ways of using Web-based tools to address support for relatives of a person with mental illness. Professional feedback may enhance the use and value of online communities.

## Introduction

Living with a person with mental illness can produce additional burdens for families [1]. Depression will rank as the second leading cause of disability worldwide by 2020 for men and women aged 15-44 years, affecting 121 million individuals worldwide [2]. The lifetime prevalence for major depression in men is estimated at 10%-13% and in women at 21%-24% [3,4]. Increasing frequencies are found among young people [5], for whom suicide is a prominent cause of death at ages 10-24 years [6], and the elderly [5].

In 40% of families living with mental illness, psychological suffering requires therapeutic interventions [7]. The family’s emotional climate can improve through family interventions [8], with lower relapse rates and better outcomes, reduced expressed emotion, and better problem-solving capacities [9,10]. Family interventions are highly prioritized in national and international guidelines, but studies show that implementation in practice has been scattered and slow. Lack of resources and stigma are barriers to the treatment of depression [2]. To optimize support for afflicted families, alternative support modalities should be explored, such as Web-based solutions.

Transportation issues, fatigue, and limited readiness to absorb information can be barriers to accessing psychoeducational programs [11]. Web-based services offer convenient access [12] and 24-hour availability of information and support, partly explaining the growth of online communities (OCs). OCs offer a space for the exchange of medical information, and provide social support and health education, entailing the benefits of major coping strategies [13]. Studies of health-related forums show an exchange of informational, emotional, esteem, and network support [14], as defined by Cutrona and Suhr [15]. Similar others can become a supporting network with important social contacts, reducing isolation and providing new perspectives [11]. Social support can be a buffering and mediating factor influencing physical and mental health [16,17]. While social isolation can be a stressor, social support can be a buffer against stress and influence health and disease processes in different directions depending on its availability and adequacy [18]. Clinical depression can be predicted by the lack of social support and depressive tendencies may reduce an otherwise healthy person’s potential for social support [18]. Families living with a person with mental illness are vulnerable and relevant support may help alleviate caregiver burden, preventing further ill health.

Research shows that expressive writing has beneficial physical and mental health effects in different user groups in several cultural settings [19,20]. Making sense of traumatic events can reduce ruminative thoughts associated with illness [21]. In a previous study [22], a Web-based tool aimed at families living with a person with depression was developed in an iterative design process that included potential users. The tool was based on a theoretical framework entailing the potential health benefits of expressive writing and social support when experiencing stressful events and showed promising results. The tool was password-protected and entailed a Web-based diary (private) and forum (users-only access). The tool promoted communication with the self and others, leading to a sense of perspective and empowerment. It promoted reflection and offered a space to ventilate feelings and share experiences, and obtain support and advice from similar others, contributing to reduced feelings of alienation and social isolation [23].

The aim of our open study was to investigate participants’ use of a Web-based tool and its potential beneficial health and psychosocial effects. Besides the diary and forum, the updated tool entailed a psychoeducation module. The tool was also targeted at an additional user group consisting of families living close to a person with schizophrenia, but these results are presented elsewhere [24]. We focused on the forum and the following research questions: What phenomena relating to the relatives’ situations stand out in the forum? What kind of social support is exchanged and with what potential effects?

## Methods

### Design

The present open study was an explorative study, including a qualitative approach to assess the forum’s value and a quantitative approach with self-rating instruments to assess caregiver burden, experiences of stigma and discrimination, and the tool’s usability.

### Intervention

**Figure 1:**
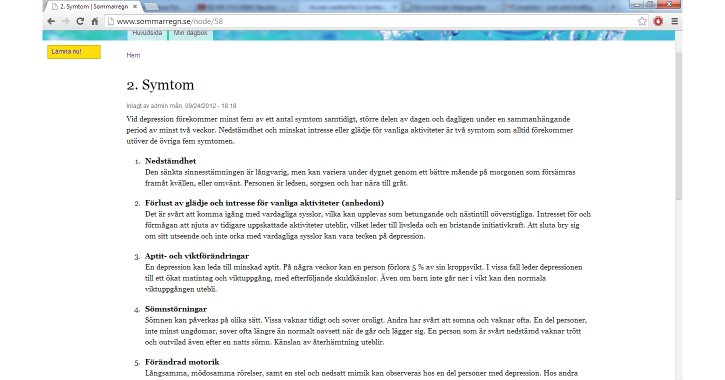
Screenshot of the psychoeducation module.

**Figure 2:**
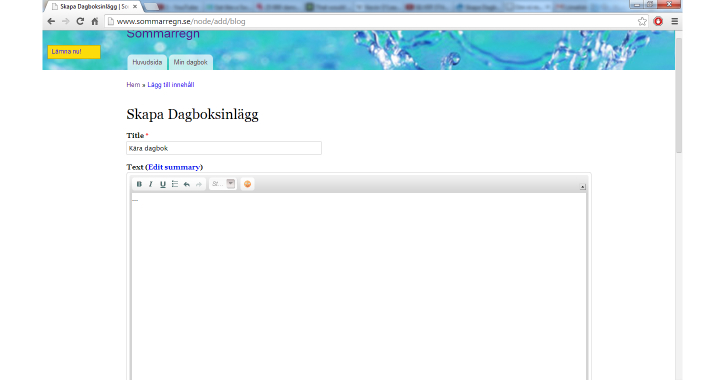
Screenshot of the diary.

**Figure 3:**
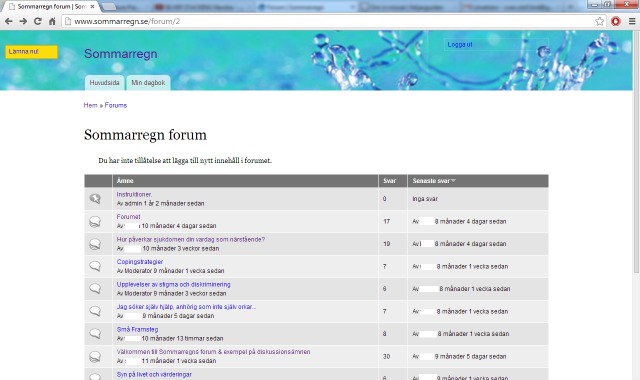
Screenshot of the forum.

The intervention consisted of a Web-based tool with three modules aimed at relatives/significant others of a person with depression: a psychoeducation module with information on mental illness, treatment, and the role of the family; a private diary, facilitating expressive writing; and a moderated and members-only forum, facilitating social support. A user peer group with patients and relatives reviewed the psychoeducation module’s contents, a novelty compared to the initial project. Access to the full website required registration, and the use of an alias and a password to protect anonymity and users’ integrity. The moderator (first author) occasionally submitted posts to spur discussions, for instance about personal needs for support and potential experiences of stigma. The test period was between February and May 2013 (16 weeks). Participants were asked to use the diary and forum weekly to ensure a certain level of activity. It was decided that participants writing posts that revealed alarming facts about, for example, signs of destructive behavior such as self-harm (in participants or patients) were recommended by the research team to seek professional help on their own behalf or on behalf of the patient (see screenshots in Figures 1-3).

### Participant Sample

Participants were recruited through advertisement in regional newspapers and on support organizations’ websites, social media, and advertisement on bulletin boards in public places (eg, libraries and hospital wards in 3 cities in southern Sweden). Inclusion criteria were being a relative/significant other of a person with depression, aged 18-80 years, having access to a computer and Internet connection, and understanding and writing Swedish. Information about the study was made available online and through email on request. Twenty-five persons enrolled by sending an informed consent form to the research team (Table 1). The sample included 6 men and 19 women, aged 18-68 years (mean, 51.80 years). No information about potential comorbidity in patients was collected.

Only 10 people wrote in the forum. Their sociodemographic characteristics appear to be representative of the total group, except for a higher mean age. For these 10 participants (9 women, 1 man), the mean age was 60.5 years (range, 48-68 years). Nine (90%) were in a relationship and 1 (10%) was single. Five (50%) shared their household with the patient, 4 (40%) did not, and 1 (10%) sometimes did. Participants’ relationship to the patient was a child (n=3, 30%), parent (n=3, 30%), partner (n=3, 30%), and other relationship (n=1, 10%). Seven (70%) had attended postsecondary school, 1 (10%) had attended high school, and 2 (20%) participants had other educational backgrounds. The majority lived in a city and half of the participants worked. Most (n=9, 90%) had previously used the Internet to search for information about depression and 7 (70%) found the information useful (n=3, 30%) or partially useful (n=4, 40%), while 3 (30%) did not find it useful. Four (40%) felt as though they received support (1 [10%] fully, 3 [30%] partially) thanks to their Internet searches. Only 2 (20%) participants had searched for/visited support groups/chats online.

**Table 1:** Background information (N=25).

Criteria		n (%)
**Marital status**		
	In a relationship	22 (88)
	Single	3 (12)
**Relationship**		
	Parent	6 (24)
	Child	8 (32)
	Sibling	1 (4)
	Partner or ex-partner	6 (24)
	Other	4 (16)
**Lives with the patient**		
	Yes	11 (44)
	No	13 (52)
	Sometimes	1 (4)
**Housing area**		
	City/township	24 (96)
	Countryside	1 (4%)
**Education**		
	Elementary school	1 (4)
	High school	3 (12)
	Post-secondary school	18 (72)
	Other	3 (12)
**Employed**		
	Yes	18 (72)
	No	7 (28)

### Data Collection and Analysis

When registering on the website, participants answered a demographic questionnaire and self-rating instruments online. The 7-item care-related quality of life for depression questionnaire (CarerQoL7-D ) [25] measures 7 dimensions (fulfillment, relational dimension, mental health dimension, social dimension, financial dimension, perceived support, and physical dimension) of caregiver burden. It also includes the care-related quality of life visual analogue scale (CarerQoL-VAS), summarizing the level of happiness with caregiver’s experiences and ranging from 0 to 10 (completely unhappy to completely happy).

The 12-item discrimination and stigma scale (DISC-12) [26] measures different aspects of stigma and discrimination related to mental illness. Nine items of relevance for caregivers were chosen from 3 of the 4 original subscales: (1) unfair treatment (6 items), (2) stopping self (2 items), and (3) overcoming stigma (1 item). Items were scored on a 5-point Likert scale ranging from 0 (not at all) to 3 (a lot) and 4 (not applicable).

After the test period, all 25 participants were provided a Swedish version [27] of the system usability scale [28], but only 13 participants (52%) replied. The scale’s 10 questions have possible values ranging from 0 to 4; the total value can be 0 to 100. Values over 70 can be estimated as good (>85, excellent), although acceptability in the field cannot be guaranteed [29]. Quantitative data were analyzed with descriptive statistics in IBM-SPSS version 21**.**

Further data consisted of forum posts, amounting to approximately 45 printed pages, including 105 forum posts/comments, and were studied using content analysis [30]. Ten (40%) participants wrote in the forum, with a range of 1 to 30 posts/comments per participant (mean, 10.5). The printouts were read several times to reach an understanding of the whole. Contents relating to the research questions were marked and coded, then grouped and abstracted into categories and subcategories. Comparisons across categories were made to identify similarities and differences. The transcripts were re-read to assess the emerging coding scheme’s fit with the material. Frequencies of diverse types of social support based on Cutrona and Suhr’s definition [15] were noted. An additional researcher (second author) analyzed data to assess the reliability of the coding schemes and results.

## Results

### Scale Summary

**Figure 4:**
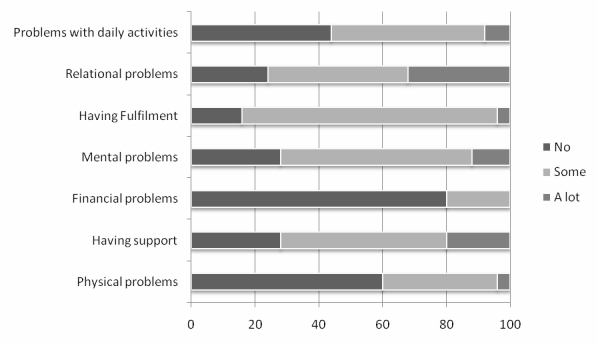
Percentages of problems/circumstances linked to the caregiving situation as reported in CarerQoL-7D.

Experiences of caregiver burden and stigma were reported through CarerQoL7-D (Figure 4) and DISC-12 items (Table 2). CarerQoL-VAS, a summary measure of the subjective burden, had a mean score of 6.16 (range, 3-10).

Results of CarerQoL showed that 84% (21/25) reported fulfillment from their caregiving tasks, 76% (19/25) reported relational problems, and 72% (18/25) reported their own mental health problems. Most (72%, 18/25) reported having support and 56% (14/25) reported difficulties coordinating daily activities with caregiving.

With DISC-12, 72% (18/25) reported having been able to use their inner strength to cope with stigma and discrimination and 64% (16/25) had concealed or hidden the person’s condition. Forty percent reported having been avoided or shunned by people who knew about the illness. Forty-eight percent (12/25) reported unfair treatment from family; 40% (10/25) in marriage or divorce and 36% (9/25) from mental health staff. Almost one-third (28%, 7/25) reported having stopped themselves from having a close personal relationship.

Participants’ subjective assessment of the tool’s usability was calculated using the system usability scale, resulting in a mean of 61.5 (range, 22.5-90; possible total value 0-100; >70=good). Most posts were written during weekdays (76/105, 72.4%) as compared to weekends (29/105, 27.6%), and between 4 pm and 12 am (64/105, 61.0%), 8 am and 4 pm (30/105, 28.5%), and 12 pm and 8 am (11/105, 10.5%).

The analysis of the forum posts resulted in 5 categories and subcategories describing areas of concern for the participants and their interactions in the forum, as described below.

**Table 2:** Results of DISC-12 (N=25).

Item		Not at all	Small	Moderate	Large	Overall (sum of small, moderate, or large)	Not applicable
		n (%)	n (%)	n (%)	n (%)	n (%)	n (%)
**Perceived stigma**							
	Have you been treated unfairly by your family?	12 (48)	5 (20)	6 (24)	1 (4)	12 (48)	1 (4)
	Have you been treated unfairly in marriage or divorce?	11 (44)	4 (16)	3 (12)	3 (12)	10 (40)	4 (16)
	Have you been avoided or shunned by people who know that you have a mental health problem in the family?	15 (60)	6 (24)	3 (12)	1 (4)	10 (40)	None
	Have you been treated unfairly by mental health staff?	12 (48)	5 (20)	2 (8)	2 (8)	9 (36)	4 (16)
	Have you been treated unfairly in keeping a job?	16 (64)	4 (16)	1 (4)	None	5 (20)	4 (16)
	Have you been treated unfairly by the police?	16 (64)	1 (4)	None	None	1 (4)	8 (32)
**Self-stigma**							
	Have you concealed or hidden your family’s mental illness?	9 (36)	4 (16)	7 (28)	5 (20)	16 (64)	None
	Have you stopped yourself from having a close relationship?	12(48)	1 (4)	4 (16)	2 (8)	7 (28)	6 (24)
**Overcoming stigma**							
	Have you been able to use your inner strength to cope with stigma and discrimination?	None	2 (8)	6 (24)	10 (40)	18 (72)	7 (28)

### Analysis of Forum Content

#### Balancing the Caregiver Role and Relationship to the Patient

##### Overview

The common ground for participating in the forum is being a relative or significant other of a person with depression. The patient’s situation is thus central and participants describe it in more or less detail. Participants partially share information about the patient’s general situation and treatment, what relationship they have to the patient, and their interaction with him/her and other family members. The situation gives rise to difficult thoughts and feelings, including wondering how to cope with the patient and the consequences of the illness on relationships and daily life.

##### Hypersensitivity

How to balance the relationship to the patient and the caregiver role stands out as a major issue for most participants. Knowing how and how much to help the ill person versus leaving him or her alone is difficult, especially with grown-up children. Finding a balance in helping a child versus encouraging independence seems difficult. Fear of severing the contact and bond with the patient makes participants reluctant to set limits, sometimes causing frustration and limiting relatives’ own lives. Participants differentiate parent-child from partners’ relationships. Parents mean that they can never let go of a child and stop worrying about his or her well-being, whereas an ill partner can be left, no matter how difficult. However, the will to support both (grown-up) children and partners and not to give up on either is strongly articulated. Participants describe their struggles and look for advice on how to find a fruitful balance. They reflect upon their interaction patterns with the patient and other family members, moving family dynamics and behavior patterns into the forefront. The nature of participants’ relationships is gently questioned by fellow forum participants to stimulate reflection. Several participants describe hypersensitivity in their relationship and assessment of the patient, leading to a constant watchfulness and perhaps premature conclusions about the patient’s status and needs. This hypersensitivity is lifted in the forum, shedding light onto the phenomenon and putting it in a new perspective for some participants.

##### A Lonesome Rollercoaster

Descriptions in the forum divulge strong feelings associated with a life with mental illness, such as sorrow, worry, fear, frustration, anger, pain, sympathy, and loneliness. The condition’s difficulties mark daily life in several ways. Participants describe patients’ self-centeredness and lack of consideration toward others’ needs as frustrating, although they realize that it is linked to being ill and possibly remorse in patients. The participants take on a caregiver role for a loved one as a natural gesture; however, prolonged periods without leisure or rest take a toll on relatives’ well-being and health. Constantly worrying about the patient’s health and life seems wearying, not the least when the person isolates him- or herself without giving any life signs, again making it difficult for participants to balance their presence in patients’ life. The fear of self-destructive behavior is looming.

Loyalty issues come out strongly through an expressed unease at mentioning troublesome aspects in participants’ relationship to the patient; however, most participants’ descriptions are suffused with empathy and caring. Participants wish the ill person the best, hoping for a brighter future. In partnerships, a growing loneliness can be seen due to the ill person’s personality change and retirement from common activities and socializing. Dark thoughts are described as contagious and participants miss the closeness and dialogue with their partner. Thoughts about the future are overshadowed by the illness. Living with the illness for extended periods and experiencing inefficient help breed feelings of helplessness and hopelessness. Participants describe losing hope countless times, but also a vital need for hope to carry on. Shifting focus from negative thoughts to the patient’s progress is suggested as a strategy to reframe the situation and avoid tunnel thinking.

#### Participants’ Own Life and Support

##### Overview

“Take care of yourself and allow yourself to live your own life” are repeated suggestions to encourage fellow forum participants to pursue own activities. Giving up leisure and social activities, to stay close to the ill person or due to lacking energy, lead to isolation and frustration. Constantly focusing on the patient’s needs and ignoring their own needs and wishes eventually affect participants’ health negatively, sometimes seriously. “Save yourself, then you can help others” catches the spirit in some of the forum posts. It appears easy to recommend, but more difficult to apply without inducing doubts or guilt. The need for space and temporary freedom from worry is obvious.

##### The Need for Professional Help

Many participants describe declining health and a need for professional help. Frustration over not being offered or even refused support is expressed. The patients’ health professionals focus on patients and refer relatives elsewhere. The content analysis indicates that participants’ focus on patients’ health and insecurity about where to ask for support complicate a potential help-seeking process, both for patients and relatives. Thinking about their health issues seems to induce guilt feelings, because participants assess that the patient is worse off than themselves. A strong need and wish for professional support is thus expressed, for example, advice on how to help the patient and themselves.

##### Coping Strategies

Participants describe diverse coping strategies, both when prompted by the moderator and spontaneously. Participants’ experiences do not necessarily change their fundamental perceptions in life, as described in the forum, but they contribute to reinforce life’s vulnerability and enhance the appreciation of certain experiences in daily life. Not making plans and taking a day at a time are described as strategies to avoid disappointment for canceling plans. Other mentioned strategies are exercise, relaxation, focusing on work, hobbies, or companion animals, spending time with friends, or traveling. Another option is to keep regular contact and help the patient in order to feel useful and in control, but also occasionally deliberately not calling the patient and hoping for the best.

#### Resources and Patient Advocacy

##### Overview

Relatives sometimes become intermediaries between the patient and the health system and other authorities. Occasionally relatives seem to become the only working link between patients and society. This can happen in emergency situations, but also to alleviate the patient’s burden, for example, by booking appointments. Dealing with the diverse organizations can be experienced as taxing, especially when problems and faulty treatments add up. Maneuvering through patient rights and administrative landscapes is energy-consuming. Participants describe both positive and negative experiences of care and how they were treated. Most express a frustration at the lack of support and at ineffective resources, both for patients and families.

##### Treatment and Participation in Care

Participants describe different experiences relating to how they were treated by health professionals. They express wonder, frustration, and anger at being excluded from care, partly relating to health professionals’ focus on patients and confidentiality rules. They comprehend the latter and don’t want to intrude on patients’ integrity, but feel at a loss for help. Participants describe a frustration at not being heard or given support when seeking care for the patient. They question the fact that family members are not automatically offered support, considering the condition’s consequences for all parties involved. Positive experiences with helpful professionals and emergency teams are also mentioned.

When sharing experiences in the forum, participants differentiate partners’ from parent-child relationships and the subsequent aptness of participation in care, although they wish for support in both cases. In the shift from youth to adult psychiatry, participants express frustration at not being automatically included in their child’s care, especially when a grown-up child cannot take care of him- or herself and depends on parents’ support. Even participants with an ill partner express a wish be included in care and supported in helping the patient. They yearn for advice on how to help and want to share their knowledge of the patient’s health fluctuations with health professionals. Despite their own professional health care experience, which some participants seem to have, they describe their role as significant others as emotionally demanding. They share their knowledge of the health system’s strengths and weaknesses in the forum, but mention needing support beyond their factual knowledge.

#### A Looming Shadow on Leisure, Social, and Professional Life

##### Overview

The illness affects daily life in many ways. It restrains leisure and socializing. Participants chose to stay with the patient when he/she feels down and sometimes isolate themselves because of low energy levels associated with the home situation. Leaving the patient alone to travel or meet up with friends seems to induce guilt feelings in many participants. The situation can affect relatives’ professional lives and economy because the psychosocial situation prevents them from working full-time. Work can also be a welcome shift of focus, although the home situation may affect the ability to concentrate. Revealing the reason for low energy levels in the professional or social network is experienced as delicate due to (self) stigma.

##### Openness Versus Secrecy

Revealing a loved one’s condition seems problematic. Worries about others’ reactions and discrimination in social and professional areas make participants cautious. They do not want others to think badly of the patient or only associate him/her with their condition. If at all most participants only mention the home situation to a few trusted friends, family members, and occasionally colleagues. Contradictory encouragements can be seen in the forum, where participants write that families should not hide and be ashamed, but rather speak up and ask for help. Simultaneously, caution is recommended in certain situations to prevent discrimination.

#### Interaction and Social Support

##### Overview

Although some participants ventilate their home situation with trusted friends, they worry about burdening them. They also wish to talk about other things than their troublesome situation and hence choose not to talk about it extendedly. Some participants describe a thinning social network due to people’s tendency to withdraw from troubled individuals. Nevertheless, when given, support from friends, family, colleagues or support groups is highly valued.

The analysis of the interaction and support in the forum shows an exchange of several dimensions of social support, including more or less equal levels of information, emotional and esteem support respectively.

##### Informational, Emotional, and Esteem Support

Participants ask about and provide informational support in the form of advice, for example, coping strategies and referrals to sources of help for patients and relatives. They exchange information on pharmacological, psychological, and other treatment alternatives and sources of support for patients and families. Participants exchange emotional and esteem support by showing sympathy and understanding of each other’s situation and by validating each other’s experiences. Participants can partially recognize themselves in others’ stories and realize that they are not alone in their situation. They support fellow participants through encouragement and by trying to convey hope. Offers of an online or offline presence, as suggested by some participants, may enlarge the social network.

##### Ventilating, Sharing, and Reflecting Give a Sense of Perspective

Reading others’ narratives gives a sense of perspective and contributes to seeing one’s situation from new angles, giving insight into diverse ways of handling the circumstances. To put experiences into words and ventilate them in the forum appear to be, directly or indirectly, beneficial. It contributes to clarify thoughts and patterns, especially when a response is provided by fellow participants. It helps them to reflect on the situation and discover new approaches. By sharing experiences with similar others in the forum, the social network can be unburdened, reducing potential guilt feelings and offering another type of support availability.

##### Empowerment, Loneliness, and Alienation

When sharing stories, some participants discover that they are not alone in their situation, partly reducing feelings of isolation. Some participants describe that they feel strengthened in their experiences and understanding of their situation when reading about others’ similar experiences. Single participants describe that they have acted or plan to do so on received advice, pointing to an empowerment process.

##### Expectations and Suggestions

The forum’s activity level is limited and the time lapse between postings and responses can be extended, which is described as a disadvantage. Sharing experiences with similar others is appreciated, however, the participants’ respective situations differ in some aspects, for example, the type of relationship to the patient, making it more difficult to relate to each other’s positions. Nevertheless, the interaction indicates an exchange of support regardless of the above. One flaming incident occurred due to the participant’s unmet expectations on the present intervention and was handled by the moderator. Some participants fear that their message will be badly received or interpreted due to the lack of physical clues, which affects how or if they write in the forum. Face-to-face contact is described as easier in that respect.

At early stages of the test period, fear of being negatively affected and further burdened by others’ stories was expressed. Participants seem to feel limited in their ability to help each other as fellow relatives without professional input. Some participants describe their role in the forum as empathic and supportive auditors. They appreciate that fellow participants take time to respond and to read about others’ experiences. Participants mention expectations and a strong wish for professional feedback in the forum. They show appreciation of the psychoeducation module, but also put forward suggestions such as cognitive behavioral therapy online, further literature tips, and increased guidance in the forum, for instance through the suggestion of specific themes or literature for discussion in the forum and professional feedback.

## Discussion

### Caregiver Balance

Caring for a person with depression can produce additional burdens [1]. Living close to a depressed person affects family dynamics and the relationship to the patient, not the least in the form of hypervigilance, as expressed in the forum. Constantly worrying, whether living together or not, can lead to a feeling of not living one’s own life, as seen in previous research [31]. Balancing relationhips seems difficult and energy-consuming. Participants yearn for advice on how to relate to and help the patient, also strenghthening previous findings [31]. In this study, 76% (19/25) reported relational problems and 72% (18/25) reported their own mental health problems, which is more than that in Flyckt et al’s (2011) study [32]. However, the latter included informal caregivers to persons with psychoses, in which a fourth lived with the patient as compared to the present 48% (11 always, 1 sometimes). A longstanding relationship, shared living with the patient, daily caregiving, and negative appraisal of caregiving are known factors that can increase experiences of burden [33]. More than half of all participants reported difficulties coordinating daily activities with caregiving, from which most nevertheless experienced fulfillment as also seen in other studies [31,32].

Although many participants received support in caregiving tasks, one-third (7/25) did not. The need for professional support for caregivers is flagrant and participants express frustration over the unavailability and inefficiency of resources to support patients and families. Lacking participation in care and not being acknowledged by professionals as an asset with valuable knowledge of the patient seem to be recurrent problems, as shown by previous research [34,35]. Participants reported experiences of stigma and discrimination from mental health staff, confirming previous research on patients’ experiences [36], but also positive encounters with attentive staff. A recent study shows that siblings of persons with mental illness do not experience openness, confirmation, and cooperation through health professionals’ approaches, leading to a sense of powerlessness and social isolation in relation to care [35].

As seen in this study, not knowing who to ask and what to ask for, for example, family interventions, may be barriers to professional help. Information and effective implementation of family interventions, known to contribute to better outcomes and lower relapse rates [9,10], are hence called for. It seems that participants’ and professionals’ focus on patients may hinder relatives’ own help-seeking process. Through participation in the forum, relatives’ experiences are validated. Effects on their own lives and health are made visible through comparison with similar others, which may become a first step in seeking support. Participants can put their experiences into words and read about others’ experiences, giving a sense of perspective and shedding new light onto their narratives. Exploring how living with a chronic disease affects daily life and storytelling have been identified as empowering methods [37], creating a sense of mastery over one’s life [38]. Besides a social network and sense of community, self-help groups offer several advantages such as the provision of role models, coping strategies, opportunities for confession, catharsis, and mutual criticism, and an antidote to a sense of being different [39].

Dealing with stigma and self-stigma are salient issues in the forum and known barriers to treatment [2]. Most participants used their inner strength to cope, but concealing or hiding mental health problems is common, confirming previous research [40]. Participants reported unfair treatment from the family and having been avoided or shunned by people who know about the person’s condition. Almost one-third (7/25) stopped themselves from having a close personal relationship, pointing to further interpersonal consequences. Fear of describing the patient in negative ways in the forum shows strong loyalty issues and guilt feelings. Choosing to keep silent may increase isolation and hinder seeking help. Being validated and feeling strengthened in one’s experiences, as seen in this study, may help break the vicious cycle and be a first step toward greater openness and lessened stigma. Participants described sharing information about their home situation with caution for fear of others’ reactions. Secrecy takes energy and isolates and assigning words to stressful experiences can help release some of the pressure [41]. In a support group like the forum, participants don’t need to fear fellow participants’ reactions; they are all gathered for the same reason, which may help reduce feelings of loneliness [35]. The lack of physical cues, a limitation with online communication, can create insecurity in interacting with others. The sender can’t see how the receiver interprets the message. Nonetheless, anonymity has been identified as one of the advantages with OCs, allowing users to come out and express themselves more freely. By meeting similar others, users’ identities can be strengthened [42] especially in stigmatized groups. As seen in this study, different types of social support, that is, informational, emotional, and esteem support [15] are exchanged, which can affect mental health positively [16,17,43]. Even if differences among group members make it more difficult to relate to each other, the same differences can give new perspectives on the situation.

The literature shows that Internet searches on mental health issues are common [44,45], with worry about someone’s health being a main motivation for seeking health information online [45]. Factors such as sex (female), age (middle-aged), education, and Internet experience (high) [45] can be associated with higher levels of such searches. This goes in line with the characteristics of the sample that wrote in the present forum. Research also shows that systems that offer a sense of anonymity can have a disinhibitory effect on seeking information online [46], which was also an essential factor in this study.

### Limitations

The sample was restricted, limiting activity levels in the forum and possibly discouraging participation. Large groups can also result in less intimacy and overwhelming message volumes [21,47]. Only 10 people wrote actively in the forum, limiting representativity. The limited participation also makes it difficult to look further into possible differences in discussion subjects in the forum. Further studies with larger samples are needed to discern potential significant trends. Identification of such themes on a larger scale may help tailor interventions depending on sociodemographic factors (eg, age, shared household) and other factors, such as the type of relationship to the patient. Nothing can be said about lurkers in this study, but lurkers can represent 80%-90% of an OC population and lurking can be associated with, for example, personal or group characteristics, external constraints, and stages of membership [48]. The moderator’s prompts may have affected the discussions’ content. Nevertheless, some prompted topics were also broached spontaneously by participants, whose responses can be interpreted as an interest in the prompts. Data were analyzed by an additional researcher (second author), strengthening the results’ reliability. The length of the test period was determined in advance. It is short in relation to a life with depression with fluctuating needs of support. Nevertheless, participants’ descriptions suggest that different stages of illness could be seen in the persons with depression throughout the test period. Recollections of diverse illness periods and subsequent needs were also described, although memories can be biased.

In this study, participants suggested professional feedback or thematic discussions in the forum. For health information to be purposeful, the needs and requirements of involved parties need to be taken into consideration [49]. Feedback on this tool can be processed and integrated into future versions to better address participants’ needs, enhancing the tool’s usability and possibly preventing further ill health and additional costs to society. Online comunities can be valuable both for exploring and addressing families’ needs and concerns, but also entail shortcomings such as delays in answering posts and unanswered questions. Further studies are needed to explore how support through OCs can be optimized, including explorations of the moderator role and potential involvment of health professionals. Studies are needed to collect evidence on Internet support groups’ relation to depression to inform decision making among concerned parties [50]. Areas for further exploration are factors influencing acceptability of and satisfaction with Internet support groups, including group size, moderation, board rules, accessibility, and naturalistic comparative studies of groups that differ in these aspects [50].

### Conclusions

Living close to a person with mental illness affects daily life and the relationship with the person with depression, including difficulties in balancing the caregiver role. Participants’ need for help in supporting the patient and themselves is flagrant. Lack of resources, stigma, focus on patients, and not knowing who to ask or what to ask for can hinder seeking help. Web-based support can help explore and alleviate the burden through the exchange of experiences and support among similar others, possibly reducing feelings of social isolation and alienation. Nevertheless, further studies are needed to optimize online support, for example, through the inclusion of professional feedback.

## Abbreviations

CarerQoL7-D: 7-item care-related quality of life for depression questionnaire
CarerQoL-VAS: care-related quality of life visual analogue scale
DISC-12: 12-item discrimination and stigma scale
OC: online community
